# Caregiver experiences and healthcare provider perspectives on managing sick young infants in primary care: a qualitative study in Awi Zone, Northwest Ethiopia

**DOI:** 10.1186/s41182-025-00873-8

**Published:** 2025-12-11

**Authors:** Tigist Getahun, Telake Azale, Mekuriaw Alemayehu, Mezgebu Yitayal, Lars Åke Persson, Joanna Schellenberg, Della Berhanu

**Affiliations:** 1 College of Medicine and Health Science, Institute of Public Health, University of Gondar, Gondar, Ethiopia; 2 Department of Health Promotion and Health Behaviour, Institute of Public Health, University of Gondar, Gondar, Ethiopia; 3 Department of Health System and Policy, Institute of Public Health, University of Gondar, Gondar, Ethiopia; 4 Health Systems and Reproductive Health Research Directorate, Ethiopian Public Health Institute, Addis Ababa, Ethiopia; 5 Department of Disease Control, London School of Hygiene and Tropical Medicine, London, UK

**Keywords:** Community-based newborn care, Health extension workers, Possible serious bacterial infection

## Abstract

**Background:**

Ethiopia implemented the community-based management of possible serious bacterial infection to improve access to lifesaving care for sick young infants aged 0–2 months. However, service utilization has been low, and the quality of care was sub-optimal, emphasizing the need to identify challenges within the primary healthcare system. This study explored mothers’ and healthcare providers’ experiences and perspectives on the management of sick young infants, including those with possible serious bacterial infections to inform policy and practice.

**Methods:**

We conducted a qualitative study including 25 in-depth and six key informant interviews with purposively selected participants, including mothers seeking facility care for their infants, health extension workers, health center staff, and supervisors of health extension workers. We audio-recorded and transcribed the interviews, and conducted inductive thematic analysis.

**Results:**

We present four major themes: caregivers’ perceptions of young infant illnesses, caregivers’ choice of place to seek care, caregivers’ experiences with caring for sick young infants at the health facility, and factors affecting the provision of quality care. Mothers acknowledged the need to seek care if their young infants became ill, although often delayed when not recognizing signs of illness, believing that it would resolve. Once identified, they had the autonomy to seek care but lacked awareness of health post services, bypassing these and seeking care at health centers, which were further away. Health extension workers viewed poor infrastructure and the perceived low quality of service as being linked to low service utilization at health posts. Mothers described long waiting times at health centers, inadequate assessment, and missing communication about their children’s conditions and treatment. Health extension workers felt they had gaps in knowledge and skills. Inconsistent availability of drugs, weak referral and feedback mechanisms, low-quality supervision, limited mentorship, and inadequate district-level ownership of newborn care constrained the delivery of high-quality services.

**Conclusions:**

This study identifies the challenges from the community to health system on sick young infants’ service utilization and quality of care at primary healthcare settings. It highlights the importance of a comprehensive approach that integrates demand-creation activities with health system strengthening efforts to ensure the consistent availability of high-quality care.

**Supplementary Information:**

The online version contains supplementary material available at 10.1186/s41182-025-00873-8.

## Background

Globally, in 2022, 2.3 million children died in their first month of life, mainly from preventable causes [1]. In sub-Saharan Africa, severe neonatal infections were the leading cause of neonatal mortality, contributing to 37% of these fatalities [2]. Many of these deaths could have been averted through appropriate diagnosis and treatment with antibiotics [3]. In line with this, the World Health Organization (WHO) recommends simplified antibiotic treatment of young infants aged 0–59 days with signs of possible severe bacterial infection (PSBI) when referral to the hospital is not feasible [4]. Many low- and middle-income countries with high neonatal mortality rates, including Ethiopia, adopted the WHO recommendation for a simplified treatment regimen of PSBI at the community and primary health facility levels [5–8].

**Fig. 1:**
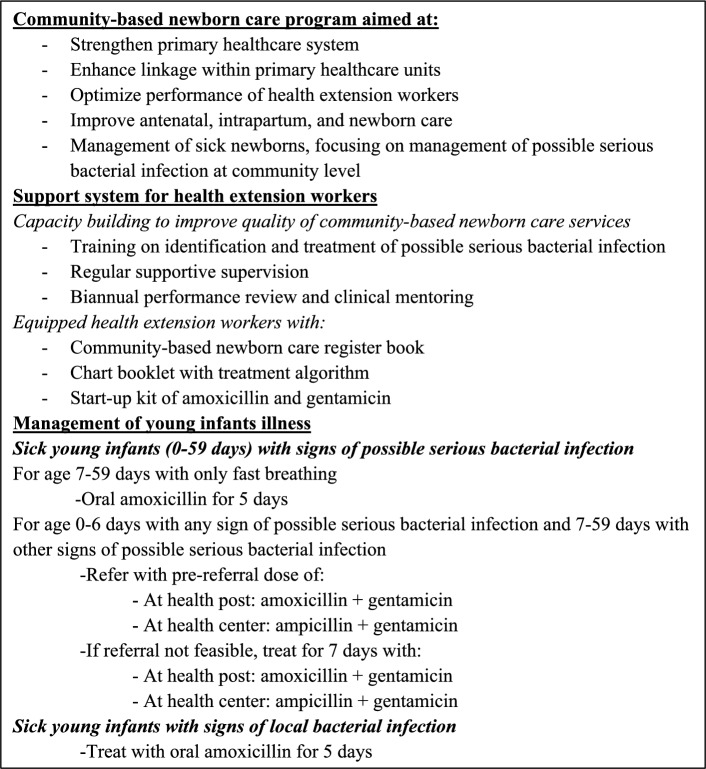
Community-based newborn care in Ethiopia

The Ethiopian primary healthcare system is three-tiered with primary hospitals, health centers, and health posts. Together, these facilities form Primary Health Care Units, each serving around 100,000 people. Each district has an average of five such units, each comprising five satellite health posts and a referral health center. To improve access to primary healthcare services, the Health Extension Program was initiated in 2003. Under the Health Extension Program, community health workers, known as Health Extension Workers (HEWs), provide basic promotive, preventive, and curative services at the community level through outreach activities and at the health post [9, 10]. In 2013, the Ethiopian government implemented and scaled up the community-based newborn care (CBNC) program under the existing Health Extension Program to improve access to lifesaving care and reduce mortality among young infants (Fig. 1) [11].

Despite these efforts, utilization of primary healthcare services for PSBI has remained low in Ethiopia [5, 12]. Reportedly, several factors have contributed to this underutilization, including community misconceptions about neonatal illnesses, socio-cultural factors, geographic challenges, limited awareness of service availability, irregular health post opening hours, drug stock-outs, and the competency of HEWs [13, 14]. Our previous research showed that many health post and health center staff did not examine sick young infants at any time in the three months preceding the survey. Only half of the HEWs and a quarter of the health center staff correctly classified PSBI. A quarter of the HEWs and a third of the health center staff treated the PSBI cases correctly [15]. Other Ethiopian studies have reported similar findings regarding the quality of care for young Ethiopian infants related to PSBI [16, 17].

While previous studies mainly focused on factors contributing to low service utilization [13, 14, 18], little is known about caregivers’ lived experiences when reaching health facilities and how these experiences shape future care seeking and perceptions of the quality of care. Furthermore, there is limited evidence on the perceived challenges in caring for sick young infants at the primary healthcare level. Therefore, this study aimed to identify mothers’ experiences regarding the care provided to their sick young infants, as well as healthcare providers’ perspectives on the care they provide for sick young infants.

## Subjects and methods

### Study design and setting

A descriptive qualitative study was conducted between December 7 and 19, 2022 in the Awi zone, Amhara Regional State, Ethiopia. At the time of the study, the Awi zone was divided into nine districts and three city administrations, with a population of 1,285,242 people [19]. There were 254 health facilities providing health services, including one general hospital, four district hospitals, 46 health centers, 203 health posts, and 190 private health facilities (114 clinics, 71 drug stores, four rural drug vendors, and one medium diagnostic laboratory center). There were 2,043 health professionals and 533 HEWs at urban and rural public health facilities [20].

The topic of this study was related to the goals of the Optimizing Health Extension Program intervention, which aimed at enhanced quality and use of services for sick infants and children at health posts in four regions [21]. However, this study was not part of the evaluation of that intervention. We identified study districts for the current study based on the number of sick young infants treated, as reported by the Awi zone health office. Three districts with relatively high numbers of sick young infants with PSBI and local bacterial infections were selected: Fagitalekoma, Dangila Zuria, and Dangila. Zonal reports were further reviewed to identify health centers that reported relatively high numbers of sick young infants with PSBI. Through this process, we identified four health centers and six health posts, which were in the health center catchment areas.

### Recruitment of study participants

We wanted to explore the quality of care provided to sick young infants with bacterial infections in the primary healthcare settings. We purposively selected participants based on predefined eligibility criteria. Participants were eligible if they were the primary caregivers of a sick young infant, who had been managed for PSBI or local bacterial infection at health posts or health centers in the previous three months, from September 1 to December 6, 2022, and resided in the facility catchment area. Healthcare providers were eligible if they had been actively engaged in service provision in CBNC or the integrated management of newborn and childhood illnesses (IMNCI) within the study area, including health center staff working in under-five clinic, HEWs, and HEW supervisors. Those who declined to participate or who were cognitively or physically unable to participate in an interview were excluded. Prior to data collection, the research team engaged with community members and local health workers to become familiar with the study context and to establish rapport and trust with the participants. At the selected health posts and health centers, registers of young infants were reviewed, to identify eligible sick young infants, and HEWs assisted in tracing the caregivers, primarily mothers, in their kebeles, the lowest administrative unit. We included healthcare providers and mothers of sick young infants who met the eligibility criteria to get different views on the quality of healthcare services provided at health facilities. The study participants were recruited from three urban and seven rural kebeles.

### Data collection procedures

We developed a semi-structured interview guide after reviewing the literature related to the main research questions (S1). The first author prepared it in English and translated it into Amharic (the local language of the study area). The guide focused on the following topics related to young infants with bacterial infection: (i) mothers’ perceptions of newborn illnesses and care seeking; (ii) mothers’ experiences of the care provided to sick young infants at a health facility; (iii) healthcare providers’ perspectives and experiences of service provision for sick young infants at health centers and health posts; and (iv) supportive supervision and provision of performance review and clinical mentoring meetings. In-depth interviews (IDI) were conducted with mothers of previously sick young infants with PSBI, pneumonia in young infants aged 7–59 days, and local bacterial infection, HEWs, and health center staff. We did key informant interviews (KII) with HEW supervisors.

The participants were contacted by the first author (TG) and a research assistant a day before the interview, and the time and location of the interview were arranged. Mothers were interviewed at their homes or a church compound. The interviews with healthcare providers were conducted at their workplace or in another preferred quiet location. All interviews were held in a comfortable location chosen by the participants to facilitate free discussion. Besides the participant and the researchers, no one else was present during the interview. After obtaining written informed consent from each participant, face-to-face interviews were conducted primarily by the first author along with a trained research assistant. The first author is currently a PhD student in public health and the research assistant has a Master of Public Health in Health Promotion and Behavioral Science and was trained on study aims, interview guide, and study ethics. The interview began with general inquiries and progressed to details based on individual responses using the interview guide. The interviews were conducted until information saturation was reached, that is until no new information emerged [22]. All interviews were audio-recorded with study participants’ informed consent, and field notes were taken during and at the end of each interview. The duration of IDIs ranged from 26 min to 1 h and 43 min, and that of KIIs ranged from 55 min to 2 h and 23 min.

### Data management and analysis

The audio-recorded interviews were transcribed verbatim by the first author and a research assistant. Transcripts were checked by the first author for completeness and consistency alongside listening to each audio record and comparing to its corresponding transcripts. Transcripts and field notes were exported to MAXQDA 2020 (VERBI GmbH, Berlin, Germany) for analysis. We conducted inductive thematic analyses consisting of reading the transcripts multiple times to get familiar with the data, understanding the participants’ perspectives, and identifying recurring ideas. DB reviewed a subset of transcripts coded line by line by TG for code consistency and credibility. Discrepancies in coding were resolved by discussion. After validating inter-coder consistency, a codebook manual was developed to assure code consistency and credibility. Thereafter, TG coded all transcripts using the developed codebook. Codes consisting of similar concepts were merged into categories and sub-categories. Categories with proximal concepts were aggregated to construct themes that answered the research questions. Themes, categories, and codes were refined and adjusted by looking for patterns, links, and contradictions within themes.

### Trustworthiness

The trustworthiness of this study was optimized using the following techniques. Experts in qualitative research and the subject matter reviewed the interview guide to ensure the quality of data and the use of simple language when conducting the interviews. We conducted data source triangulation from multiple participants to get a more profound and broader spectrum of perspectives. These participants included mothers of previously sick young infants from rural and urban settings and various healthcare providers, such as health center staff, HEWs, and HEW supervisors. Peer debriefing was done with researcher team from field work to drafting of findings. To ensure dependability, the selection and recruitment of participants, data collection methods, and analysis processes were described in detail. In addition, representative verbatim quotations were used for each theme to enable readers to evaluate the interpretation of findings.

### Reflexivity

The first author, a female researcher, holding a bachelor’s degree in public health and a master’s degree in emergency obstetrics, gynecology, and general surgery, conducted the data collection and analysis. Her background in maternal, neonatal, and child health, gained from working in both health centers and hospitals, provided a solid understanding of the healthcare system, the subject matter, and helped her establish trust with participants. She spoke the local language well, which facilitated communication and rapport. She was aware that her professional perspective might influence data interpretation. To minimize potential bias, she maintained reflexive field notes and regularly reflected on her assumptions and interactions throughout the research process. In addition, the last author independently reviewed a subset of transcripts, and coding discrepancies were resolved through discussion, functioning as a peer debriefing to ensure the analysis remained grounded in participant data. Furthermore, all authors had previous experience in qualitative research studies.

## Results

### Study participants characteristics

We reviewed the integrated Community Case Management (iCCM) and IMNCI registers of children aged 0–59 days at health posts and health centers, respectively. Among the selected six health posts, only one sick young infant had been seen in the preceding three months. At the four health centers selected, we identified four cases with PSBI, ten cases with rapid breathing classified as pneumonia among young infants aged 7–59 days, and 11 cases with local bacterial infection recorded in the preceding three months. Among those, we interviewed two mothers of young infants with PSBI, four mothers of young infants with pneumonia, and five mothers of young infants with local bacterial infection. We also interviewed HEWs and health center staff serving these households. A total of 25 IDIs and six KIIs were conducted. The age of mothers with sick young infants ranged from 23 to 43 years, and most had given birth at health facilities (Table 1).

**Table 1:** Socio-demographic characteristics of 11 mothers in Awi zone, Amhara regional state, Ethiopia, 2022

Variable	Category	*n*
Age	20–29 years	7
	30–39 years	3
	≥ 40 years	1
Educational level	No formal education	3
	Primary	4
	Secondary	3
	Degree	1
Place of delivery	Health facility	9
	Home	2
Residence	Rural	8
	Urban	3

The HEWs’ work experience ranged from 5 to 16 years. Most health center staff had a Bachelor of Science degree in nursing. They had less than five years of working experience in under-five clinics. The majority of HEW supervisors had fewer than five year experience of supervision, with most having less than three years (Table 2).

**Table 2:** Socio-demographic characteristics of healthcare providers in Awi zone, Amhara regional state, Ethiopia, 2022

Variable	Category	In-depth interviews		Key informant interviews
		HEWs *N* = 8	Health center staff *N* = 6	HEW supervisors *N* = 6
		*n*	*n*	*n*
Age	20–29 years	4	4	2
	30–39 years	4	2	4
	≥ 40 years	0	0	0
Educational level	Level III(Certificate)	2	–	–
	Level IV(Diploma)	6	2	4
	Degree	–	4	2
Profession	HEW	8	–	–
	Nurse	–	4	5
	Pediatric nurse	–	1	0
	Health officer	–	1	0
	Environmental health	–	0	1
Years of service (total)	< 5 years	0	0	1
	5–9 years	1	4	4
	≥ 10 years	7	2	1
Service years at current position/site	< 5 years	5	4	5
	5–9 years	2	2	1
	≥ 10 years	1	0	0

The findings of this study are presented with four major themes. These are: (1) caregivers’ perceptions of young infants’ illnesses; (2) caregivers’ choice of place to seek care; (3) caregivers’ experiences of sick young infants’ care at a health facility; and (4) factors affecting the provision of quality care. The four major themes with ten sub-themes are presented below (Table 3). The sub-themes occasionally overlap, since the analysis combined caregivers’ and healthcare providers’ insights to present both concurrent and contrasting perspectives on the same phenomena.

**Table 3:** Summary of themes and sub-themes on experiences of caregivers and healthcare provider perspectives on the management of sick young infants in Awi Zone, Amhara regional state, Ethiopia, 2022

Themes	Sub-themes
Caregivers’ perception of young infants illnesses	Illness recognition Decision to seek care - Perception of severity - Young infants’ illness progression - Autonomy to seek care
Caregivers’ choice of place to seek care	Service utilization at health posts - Awareness on availability of curative services at health posts - Infrastructure and closure of health posts - Perceptions of the quality of services at health posts
Caregivers’ experiences of sick young infants’ care at a health facility	Waiting time to receive care Quality of healthcare providers’ assessment of sick young infants Level of communication between healthcare providers and caregivers Caregivers’ satisfaction and their recommendations to others on seeking care
Factors affecting the provision of quality care for sick young infants	Healthcare provider level - Providers’ knowledge, skills and confidence - Motivation and commitment Health facility level - Availability of drugs, guidelines and equipment Health system level - Referral and feedback system - Supervision and clinical mentorship - Ownership and sustainability of the newborn care services

### Caregivers’ perceptions of young infant illnesses

1. **Illness recognition**

Mothers acknowledged that if their young infants became ill, they should seek care at a health facility. They reported that healthcare providers had taken the opportunity during facility delivery or immunization days to inform them to seek care if their infants got sick. However, when their young infants were ill, some mothers could not recognize the illness signs.

*“I didn’t know earlier [as the baby was sick] when she became irritable. But when I understood I took her [to the health center] immediately. I realized that she had been ill for a while, and her irritability was due to her illness, but I didn’t recognize that.” (Mother, 29 years, IDI).*

Another mother, whose young infant had pus on his umbilicus, also told:

*“He was not sick; it was just that it [the pus on umbilicus] wouldn’t get dry… I was expecting it should dry within a few days, I didn’t know. We thought it was wet due to being a newborn, I didn’t know it will be like this. I did not think it was a problem.” (Mother, 30 years, IDI).*

Healthcare providers also said that mothers had difficulties in recognizing newborn danger signs. One health center staff shared his experience:

*“You know what one mother did once, when I asked her if her baby has fever and she said no. When I measured his temperature, I found it was 39.6 [*^*o*^*C]. Since then, I don’t believe people unless I measure myself. Every time a baby comes, I stand there with a thermometer; it is a must.” (Health center staff, IDI).*

Overall, mothers reported that their young infants had one or more of the following signs: cough and vomiting, reduced breastfeeding, umbilical area complaints (pus, redness, swelling, protrusion, bleeding, and wetness), fever, weakness, difficulty in breathing, fast breathing, irritability, and bloating.

2. **Decision to seek care**

Most mothers did not seek care immediately after recognizing some signs of illness. Most reported seeking care around two days after their young infant showed signs of illness, while others sought care after even more time. Factors affecting their care-seeking are detailed in this section.

***Caregivers’ perceptions of the infant’s illness severity***

Most mothers perceived that their young infant’s illness was not severe and would resolve on its own. This was due to their perceptions of causes of illnesses. For example, they perceived that redness or pus on the umbilicus was due to the umbilicus being pulled by healthcare providers during delivery. They referred to cough, fever, or fast breathing as ‘*berd*’ (exposure to cold weather) and common cold (cough and fever).

*“I thought it was a common cold and I thought it was simple. I didn’t think it would get worse. You see, since she is small it weakened her within a day. After she became sick, on the third day the illness worsened and it just took 20 minutes to weaken her when I came back from the baptism of her twin brother.” (Mother, 24 years, IDI).*

Even after recognizing that the illness was severe, many of the mothers delayed seeking care expecting the illness to resolve on its own.

***Young infants’ illness progression***

Mothers reported that they sought care for their sick young infants at a health facility after days of delay. Their main reason for seeking care was the worsening condition of their young infants. Some of the newborn care practices at home may have inadvertently worsened the condition.

*“I thought he [baby] would improve when I bathed him with cold water, and I bathed him with cold water consecutively but without improvement. However, he got worse. Then I said let us go and we went [to the health center].” (Mother, 28 years, IDI).*

***Autonomy to seek care***

Mothers of sick young infants stated that, aside from themselves, fathers, grandmothers, and neighbors were involved in the decision to seek care. Most mothers made the final decision to seek care. When asked who made the final decision, one mother stated:

*“It was me, his mother [smiling], it was me. I suffered a lot to get him and since I didn’t want him to get hurt, I was the one who took him [to health center]. Even though my mother told me that he will be fine, I said he should be treated and I made him to get the treatment.” (Mother, 38 years, IDI).*

Another mother also said:

*“Erree [an exclamation] I didn’t consult anybody. It was me who took her [the baby] to the health center.” (Mother, 29 years, IDI).*

Though the final decision was made by mothers, the involvement of community members had an impact on timely care seeking.

*“She was very sick at that time; I didn’t sleep for 2 or 3 days. I just went [to the health center] on the 6th day of her illness. I waited because they [neighbors] said she will improve…” (Mother, 25 years, IDI).*

### Caregivers’ choice of place to seek care

1. **Service utilization at health posts**

When mothers finally decided to seek care, they mainly sought care at a health facility. Almost all mothers bypassed the health posts and sought care at health centers. According to our register review, only one young infant was seen at six health posts in the three months preceding this study. Therefore, cases were exclusively from health centers. Nevertheless, the factors for low or no utilization of newborn care services at health posts are described below.

***Awareness of availability of curative services for sick young infants***

Some mothers indicated that they would prefer to primarily seek care at a health post, given its proximity to their homes. This would avoid incurring transport cost and facilitate communication with the HEWs. However, most mothers reported not being aware of the availability of curative services for sick young infants by HEWs at the health posts.

*“Since they [HEWs] did not inform us to bring children when they got sick, how could we go there. We go to higher facilities. That is why we didn’t go there. If the materials were available; this is near for us rather than going there to town and struggle.” (Mother, 28 years, IDI).*

This unawareness was despite knowing the availability of immunization and curative services for children aged 2–59 months at health posts.

*“Ayee [No] it is for above two months. For under-two months what will they give? There is no service except home to home counseling on hygiene and to start immunization.” (Mother, 27 years, IDI).*

***Infrastructure and closure of health posts***

Some mothers reported that health posts were usually closed and HEWs were absent from the health posts. A mother who went to a health center explained the reason why she did not go to the health post first:

*“We go to the health center, we don’t go to them [HEWs]…Even when women go for family planning, they will not find them [HEWs] on working days. They usually will not be available at health posts. They sometimes will be found [at health posts] on monthly holidays like ‘Mariam’ or ‘Balegeziyabeher’ [Christian holidays that will be celebrated on 21th and 29th], but if we go today or tomorrow, we won’t find them…” (Mother, 27 years, IDI).*

HEWs also agreed that health posts may be closed due to shortage of staff, work overload, or being engaged in other activities like health campaigns.

*“When there are emerging activities the health post will be closed. We will close it for example when there is a 10-day campaign. During health insurance [launching], we usually work on and off and it will be closed for around two months. When we go to training, or when we are out at the same time it will be closed…” (HEW, IDI).*

Most HEWs reported that the health posts were not conducive to provide curative services to sick young infants. Health posts lacked adequate space and were not clean and attractive for managing young infants. One of them described:

*“You have seen the health post. It doesn’t seem as a place to provide treatment; it seems like a house of cows. I think it is due to this that mothers do not come…the building is poor, as to me I don’t think storing medicine here and giving to children will cure them…” (HEW, IDI).*

***Perceptions of the quality of services at health posts***

As stated earlier, some mothers preferred to receive care first at health posts if equipment and medicines were available. However, most HEWs perceived that mothers did not seek care at health posts due to their perceptions of more skilled healthcare providers at higher level facilities, poor functionality of the health posts, lack of trust and confidence in HEWs´ ability, and the referral of all sick young infants by HEWs regardless of their clinical presentation.

*“They don’t come to us. They go to the health center. I think our place, the health post, is not a convenient place. Anyway, it is easy for them to go to the health center, they have a habit of getting treatment by better healthcare providers. They think we don’t have drugs. They think there is no drug for newly born babies. I think it is like that and since it is free here [health post] and there [health center] they prefer a better treatment.” (HEW, IDI).*

### Caregivers’ experiences of sick young infants care at a health facility

Mothers were asked about their experiences of care for their sick young infants at health centers. They described long waiting time to see a healthcare provider, how their baby was examined, the medication they were provided, and their level of satisfaction and recommendation of care to others. These experiences are detailed in this section.

1. **Waiting time to receive care**

Most mothers had waited for a long time after arriving at the health center before their sick young infants were seen by a healthcare provider. They said this was due long queues and poor service provision system at the card room, which is a place, where patients’ records were kept.

*“As I told you, we went there in the morning. We thought they will start at 2:30, and we arrived there at 3:00. The baby was with my mother. I went to the card room and I had waited a very long time, it was a very long hour, there were many people and also long queues. At the card room they leave and come back as they want [card room staff], they don’t read names properly. Due to these problems, I think the card room has taken a long time for me.” (Mother, 23 years, IDI)*

2. **Quality of healthcare providers’ assessment of sick young infants**

Mothers reported that the healthcare providers did not systematically examine and assess the young infants. Most mothers indicated that the healthcare provider merely asked them about the main complaints and did not ask about any relevant medical history. Nearly all mothers stated that no physical examination was done.

*“I don’t know. It might be that they didn’t have the instruments. They didn’t do any examination. They asked if there is fever but didn’t measure her temperature. ‘How is her breathing? What makes her sick? Where is her illness? Is it in her chest or abdomen?’ They didn’t think like that; rather they just asked us. We went there because we thought they are doctors and they know. However, they asked us what is wrong; they didn’t consider finding the disease by examining with an instrument.” (Mother, 27 years, IDI).*

This problem compromised their trust in the healthcare system.

*“Their provision without examination is not good, it will hurt. The medicine will not match the disease; it might hurt them [babies] inside. So I would be happy if they did an assessment. Now since they don’t make any examination even though she is sick I don’t want to go [to health center]. I can buy [the medicine] from a private pharmacy by telling them the symptoms.” (Mother, 25 years, IDI).*

The provision of treatment without proper history taking and physical examination was also acknowledged by the healthcare providers. This was attributed to lack of training, poor commitment to work, provision of care by untrained staff, and case overload.

*“When there is patient overload, increased number of services, and lack of attention you might not give quality service. You might manage severe [classification] as normal, so attention should be given. Under-five and under-two months are in one room and this facility serves around 45 cases, minimum 30 cases per day. When there is a high load, I suspect there might be lack of attention… When there is such kind of load; we might not observe and examine.” (Health center staff, IDI).*

3. **Level of communication between healthcare providers and caregivers**

Most mothers said there was a lack of information on the type of illness and no counseling on how to manage their young infant’s illness at home. They also reported that the healthcare providers did not inform them on the dose, frequency, duration, or side effects of the prescribed medicines. Such incomplete information compromised adherence to the prescribed medicine.

*“The medication given was a syrup. I gave him for 5 days. I didn’t ask for how long, I just assumed once it is open it won’t stay long so I stopped it after giving him for 5 days. No one told me for how long it should be given…I gave him three times a day: in the morning, at mid-day, and at night, and the amount I gave him was little. It was just two drops.” (Mother, 38 years, IDI).*

The lack of counseling led mothers to seek alternative advice, which could be misleading. One mother who had sought care at a health center and received insufficient counseling expressed:

*“When I called [phone] the hospital, he [the healthcare provider] told me ‘her [the baby] bloating was because her capacity is small. Since she has minimal capacity, don’t breastfeed her much. Rather breastfeed her in small amounts’. When I gave her like that [minimizing amount of breast feeding] she had minimal change. The information I get from the hospital was from the one who saw me for ultrasound. …she became fine only by the advice I got. She didn’t improve by the medicine given without assessment. Rather, she improved by the advice.” (Mother, 25 years, IDI).*

4. **Caregivers’ satisfaction and their recommendations to others on seeking care**

Most mothers were satisfied with the care provided to their sick young infants despite their experiences of long waiting time, lack of examination, inadequate counseling on medication, and even lack of medication. The reason for their satisfaction was the fact that the health of their young infants improved after receiving treatment. Although mothers highlighted various service-related problems, they still described the care as satisfactory, since their young infants recovered following treatment.

*“I was happy; the main thing was the response [improvement after treatment]. I was even happy for all. I felt frightened when he said it [medicine] was not available, whatever either here [health center] or there [private pharmacy] I was happy.” (Mother, 30 years, IDI).*

However, one mother reported being dissatisfied:

*“I was not satisfied, I was not happy. Since there was no improvement I was not happy. I was dissatisfied with their treatment; they have a problem with management. They didn’t do examination; they just asked me…They shouldn’t prescribe medication only by asking me, what does that do? I might have mistaken her illness when I became shocked at that time and if they gave her the medication with that, it might hurt her. For that, I am dissatisfied and I will be happy if they fix it.” (Mother, 25 years, IDI).*

All mothers said that they would recommend facility care for neighbors and families for a sick young infant in the community due to their newborns’ improved health after treatment.

### Factors affecting the provision of quality care for sick young infants

In this section, we present factors affecting the provision of quality care for sick young infants at health worker, health facility and health system levels.

1. **Healthcare worker level**

***Providers’ knowledge, skills and confidence***

Health center staff reported that they had no difficulties in identifying PSBI in young infants and they referred all young infants with PSBI to hospitals, as recommended by the guideline. The enabling factors raised were being trained on IMNCI and the use of guidelines.

Most health center staff had been trained five years previously and they had not received any training updates. They had received seven days of training with theoretical and practical sessions, which helped them to identify, classify, and treat young infants according to the guideline. They acknowledged the impact of training on their performance.

*“After I was trained and before I trained—there is a difference in my work because I understand that I used to treat without knowing. After I become trained, I never work without using the chart booklet. I am working according to the guideline.” (Health center staff, IDI).*

In contrast, most of the HEWs were trained 10 years previously. HEWs reported forgetting how to manage sick young infants and feeling loss of confidence in their skills. One HEW explained:

*“There is a knowledge gap, let alone this long-time gap [training gap], we should have regular training updates. Now I have forgotten it [managing PSBI]…” (HEW, IDI).*

Although HEWs reported providing postnatal care and immunization, most reported not encountering a single sick young infant in the past three months, highlighting the limited opportunities to practice and refine their case management skills. A health center staff also commented on HEWs’ knowledge:

*“Let alone the classification of very severe disease, there is even a problem with identifying and treating local bacterial infection because of limited capacity. Even a baby with umbilical wound [pus, redness] will not be treated correctly.” (Health center staff, IDI).*

All HEWs, health center staff, and HEW supervisors strongly emphasized the need for refreshment training or training updates.

***Providers’ motivation and commitment***

The health center staff raised the shortage of trained staff as a concern. This resulted in the existing staff remaining in the under-five clinic for long periods, leading to burnout. One health center staff explained:

*“The number of trained staff should be increased so a person should work in rotation without being fed up. There are excess cases of working at the same location [under-five clinic] for 5 years. You can guess how much burden it might be.” (Health center staff, IDI).*

Most HEW supervisors agreed that HEWs had a good level of commitment and motivation. However, one HEW supervisor stated that HEWs had poor commitment:

*“When there is a mother who delivers at home accidentally or after she is discharged [from health center] after 24 h, they [HEWs] don’t follow-up the mother before 72 h after delivery according to the schedule due to carelessness. They forget as they should visit up to ten houses and if they [HEWs] only stay at the health post, they [mothers] won’t come.” (HEWs supervisor, KII).*

The heavy workload and involvement in multiple activities reduced the HEWs’ focus on infant care and contributed to burnout.

2. **Facility level**

***Availability of drugs, guideline and equipment***

Health centers were equipped with the necessary equipment and updated guidelines. However, the inconsistent availability of drugs was a problem.

*“In our facility to treat VSD (very severe disease) first starting from the commodities [drugs], it is advised to treat by injection and we don’t have the commodity [drug], even to refer after giving the first dose of ampicillin and gentamicin. There are conditions when these might be unavailable.”(Health center staff, IDI).*

Most mothers expressed their experience of unavailability of the prescribed drugs at health centers. A mother whose young infant was referred to a hospital described the reason for her referral:

*“[The healthcare provider said] ‘Her fever is high; we don’t have a drug to give.’ She was little, she was not even 40 days [old], she was 39 days [old].” (Mother, 43 years, IDI).*

There was also a lack of equipment and updated guidelines as well as inconsistent availability of drugs at the health posts.

*“Now, if we take commodities and instruments at the health post, it is difficult to say it is available. It lacks many things. We will bring drugs if it is available at the health center. If it is not available we will refer [young infants] to them [health center]. Many things are lacking at the health post… For example, we don’t have gentamicin here. We don’t have it to treat under-two months. It has been a long time. Even when we went to health center to request, they told us even they don’t have it. It is not available.” (HEW, IDI).*

3. **Health system level**

***Referral and feedback system***

All interviewed health center staff stated that young infants with a diagnosis of PSBI were initially referred to hospitals and only treated at the health center if referral was not possible. Accessibility of hospitals and availability of ambulance service allowed young infants to be managed at hospitals. Providers mentioned that mothers were also willing to accept the referral if provided with information about the illness, the need for hospital-level treatment and the unavailability of instruments and drugs at the health center.

*“‘It is beyond our capacity. The baby should be treated at the hospital. He needs these things. These necessary things are not available here but are available at the hospital and he should go and be treated at the hospital.’ When I told her that she [the mother] knows we don’t have drugs here, and if it is not available, we can’t bring it from anywhere. So, if that is so, if you don’t have drugs, if you don’t have equipment [instrument] we will go. There is no problem anyways. Send us there, soon… It is no problem’. Some mothers will say like that.”(Health center staff, IDI).*

Even though mothers appreciated that some illnesses required referral, they recommended equipping health centers and providing the service near their home to avoid financial costs and exhaustion of travel associated with referrals.

At the health post, some HEWs indicated that when, on rare occasions, mothers came to them for their young infant’s illness, they were referred, which affected care seeking at health posts.

*“The reason they don’t come here [health post] is that there is not much service as for children [2–59 months]. Since we see and refer them, they directly go to the health center.… Even if they come, we mostly refer them. There is no curative care at the health post due to unavailability of drugs for less than two months.” (HEW, IDI).*

The study participants raised their concern about the week referral-feedback system. At health centers, although referral was made with a referral note, formal written feedback was not provided from the hospital. One health center staff explained the importance of feedback:

*“The feedback is the one that makes me decide if I am right or wrong about the baby I referred. If that was not sent I may have made a mistake. However, if the feedback system is strengthened I may know my mistake and correct the next time. If I did an unnecessary referral, I will learn from it and if I referred appropriately I will also learn. I will learn from the right and wrong things.” (Health center staff, IDI).*

HEWs sent young infants without any written referral slip irrespective of the case.

*“Actually I didn’t send her [the sick baby] with referral paper. I just told her [the mother] to take her [the sick baby] directly to the health center.” (HEWs, IDI).*

***Supervision and clinical mentoring***

The health center staff had irregular supportive supervision from district and zonal health offices. Most supervision was said to be encouraging, while it sometimes lacked depth. The staff suggested more frequent supportive supervision by knowledgeable personnel.

At health centers, the HEWs supervisors mentioned there was weekly supportive supervision to health posts by an integrated team from the health center engaging staff from under-five, maternal, out-patient, laboratory and pharmacy departments. Most HEW supervisors had no training on CBNC or how to provide supportive supervision. In addition, half of them had no exposure to working in under-five clinics. Most supervisors reported that they had no difficulty in supervising; however, one HEW supervisor pointed out an issue:

*“…Since I didn’t get the training, I can’t give them the direction assertively. Since the work of the HEW and HEW supervisor is similar, if we get the training together it would help us to show the weakness or gap. There are times when I went to provide support and yet got the support from them [HEWs]…” (HEWs supervisor, KII).*

Most HEWs received quarterly supportive supervisions. Previously, when non-governmental organizations supported the CBNC program, the supervisory visit involved checking the young infant register book for completeness, adherence to guidelines with respect to assessment, classification, treatment and follow-up dates, correcting identified gaps, and encouraging their good performance. However, the HEWs felt that the current supportive supervisions did not fill the gaps and lacked focus on young infant care.

“*I don’t think it will fill any gap, because they come [to supervise] by integrating other activities too and ask ‘is there any [treated young infant] or not, did you treat or not’… I don’t think this will improve our skills unless they ask us why we didn’t treat any [young infant]. I don’t think this [asking on numbers] will advance our skills…I can’t say checking the register and saying ‘did you treat or not’ will fill gap or advance individuals’ skills.” (HEW, IDI).*

They suggested having separate under-five supportive supervision, which could include on-site refreshment training and equipping the health posts with necessary equipment and drugs.

Although HEWs wanted supervision and there was a committed integrated supervisory team at health centers, supervision was further hindered by poor transportation, such as lack of vehicles and long travel distance.

The biannual performance review and clinical mentoring meetings were reported to have been discontinued. Almost all HEWs reported that it had been four years, since they last participated in a performance review and clinical mentoring meeting. Even though they had forgotten the details of the meeting, they acknowledged its impact on their skills, performance, and motivation.

*“It created motivation to work. I mean they compared one health post with the other based on the number, quality of care and this will encourage and give moral. It created motivation to work. It created [motivation] for us and it created [motivation] for me.” (HEW, IDI).*

***Ownership and sustainability of newborn care services***

The healthcare providers mentioned that there had been support from non-governmental organizations in the past. There were trainings for healthcare providers and community leaders, regular supervision, clinical mentoring, renovation of health posts, and mobilization of communities on young infants´ care seeking. They acknowledged that more young infants came for treatment at health posts and health centers during that time. However, they felt that when the district health office had taken over these activities, the sustainability and ownership of newborn healthcare services was not at the expected level. One health center staff pointed out:

*“…Attention needs to be given as before to the treatment of babies since it is a forgotten program. If proper attention is given, if support is available it would benefit children…As I told you before, when there are NGOs, ha, ha, ha (laughing), the number of people participating is high. When the NGOs are phased-out, the people will also phase-out and the work will also phase-out.” (Health center staff, IDI).*

Most HEWs also described that their lack of attention to young infant treatment at the health post was due to inadequate supervision and monitoring, thus they focused on activities, where they would be monitored.

*“On monthly reports, it should be a must if we were also asked the report of under 2 months [old] rather than merely asking above 2 months [old] children treatment report. Now it is forgotten, there is not much. If there is a patient, we will write [report] if there is none, it is zero. It will be good if they ask us to bring [reports of treated young infants] during supervision…It is like that, they don’t follow-up much. I even start to remember when you ask me and I felt [bad] for that.” (HEW, IDI).*

## Discussion

We have shown that Ethiopian mothers with sick young infants delayed seeking care because of poor recognition and interpretation of young infant signs of illness. There were perceptions that the illness was not severe and would resolve on its own. Mothers bypassed the health posts and went to health centers, which were further away, due to a lack of awareness about the health post curative services, or were forced to do so due to the frequent closures of health posts. The HEWs reported that poor infrastructure and perceived low-quality services were the reasons for low service utilization at health posts. At health centers, mothers experienced long waiting times, inadequate physical examinations of their sick young infants, and inadequate communication about their young infant’s disease and treatment. Nevertheless, most mothers expressed satisfaction with the care they had received, because their young infants’ health improved. HEWs felt they lacked the knowledge and skills to manage PSBI, as well as low motivation and limited support. Several barriers existed to providing quality services, including the absence of refresher training, irregular and low-quality supportive supervision, discontinued clinical mentorship, drug stock-outs, weak referral and feedback system, and weak district-level ownership of newborn care services.

Caregivers need to be supported to understand when, where, and how to access care and to act promptly when required [23]. We found that mothers recognized the need to seek care for a sick young infant. Mothers could decide to seek care despite dissenting advice from families and neighbors. However, mothers often failed to recognize signs of young infant illnesses, which resulted in delayed care-seeking. This finding is consistent with previous studies in Tanzania, Ethiopia, Uganda, Nigeria, and Indonesia [23–27]. Even after recognizing young infant illnesses, mothers often attributed them to less severe causes, such as birth-related factors or mild discomfort that was likely to resolve spontaneously. They sought care when the young infants’ illnesses were getting worse, as also reported in previous African or Asian studies [13, 14, 25, 28]. This finding emphasizes the need for targeted strategies to improve caregivers’ ability to recognize newborn danger signs and the importance of timely skilled care seeking. Continuous education through postnatal counseling and media could enhance awareness [29, 30]. Furthermore, engaging family members and community influencers to support mothers’ decision on prompt care seeking may also be crucial [31].

We found that mothers bypassed health posts and sought care at health centers, being unaware of the availability of curative services for sick young infants at health posts. Even when aware, participants reported challenges such as the health posts being closed and HEWs frequently absent, mainly attributed to a shortage of staff, excessive workloads, competing tasks, and low commitment among HEWs. Furthermore, HEWs reported insufficient knowledge, skills, and confidence in managing PSBI in young infants. Factors that could improve HEWs’ skills in managing PSBI in young infants, such as refresher training and clinical mentoring [11, 30], had been discontinued and supportive supervision [5, 32, 33] was infrequent. Even the existing supervision lacked depth, focused on coverage rather than content and was at times provided by unqualified staff. These findings highlight the need to strengthen community empowerment and demand generation initiatives through HEWs community dialogue [10, 34] and improving HEWs’ capacity through high-quality supervision focused on a supportive and problem-solving approach [35].

Short waiting times, clear communication, respectful care, and proper counseling are elements of good quality care [36]. Mothers experienced delays in receiving care and poor communication from healthcare providers, including inadequate medication information. These factors compromised adherence to medications, potentially leading to antibiotic resistance. Poor provider–client communication compromises compliance and trust in the healthcare system [36]. Despite these problems, mothers were generally satisfied with the service their young infants had received as they recovered from their diseases. Therefore, measurement and interpretation of patient satisfaction as a measure of quality needs caution [36].

The IMNCI guideline indicates that sick young infants should be assessed not only for the presenting signs and symptoms but also for other disease conditions, including signs of PSBI and local bacterial infection [37]. However, in this study, sick young infants receiving care at health centers did not receive the recommended assessment, suggesting providers’ poor compliance with the recommended clinical practices. We have also previously shown that only half of HEWs and a quarter of health center staff correctly classified PSBI cases. Treatment for these conditions remained suboptimal at both facility types [15]. Other Ethiopian studies have also shown low levels of compliance to the IMNCI guidelines [16, 38]. Similar to other studies, we found that case overload, lack of training and burnout could have contributed to poor compliance with the recommended clinical practices [39, 40]. These challenges suggest the need to avail sufficient trained staff along with high-quality supportive supervision and mentoring to ensure compliance with the clinical guidelines.

The referral pathway for young infants with PSBI involves referring cases to higher facilities with a pre-referral dose of antibiotics, and treatment is provided at the health post or health center level only when a referral is not possible [41]. In this study, all PSBI cases at health centers were referred to hospitals, reportedly due to the severity of the young infants’ illnesses and the lack of facility equipment and drugs. Under these circumstances, mothers accepted the referral. Although referral acceptance is promising, as the WHO recommends, more efforts are needed to strengthen health centers to provide treatment when referral is not feasible [32, 33]. In addition, in accordance with previous studies [15, 17, 42, 43], we reveled that inconsistent availability of antibiotics at health posts and health centers are challenges. These challenges contradict the main aim of CBNC program of managing PSBI at community and referral health centers level to increase survival and access to lifesaving care. Hence, continuous efforts are needed to strengthen the supply chain system, enhancing the availability of these drugs [43, 44].

Healthcare providers reported that district-level ownership of the newborn care program was weak after NGOs handed over activities to the district health office. At the initiation of the CBNC program, NGO partners played a crucial role in training healthcare providers and community leaders, mobilizing communities to seek healthcare for sick young infants, providing supportive supervision, conducting performance reviews and clinical mentoring meetings, and distributing CBNC supplies and antibiotics [45, 46]. We only have the healthcare providers’ perspectives, and district health managers’ experiences and perspectives would have been valuable. Previous studies have highlighted weak ownership and poor integration of PSBI services within the district health system [47]. This implies strengthening district-level ownership is crucial for achieving the intended aim of managing PSBI at the community level and further reducing neonatal mortality.

The current study explored the care process from the perspectives of healthcare providers and mothers. Understanding users’ experiences of care is essential in efforts to improve care. We also reported on providers’ perspectives, thus using data triangulation to gain a deeper understanding of the context. One limitation is recall bias, although we used a three-month period to minimize this. We did not include the experiences and perceptions of the district-level health managers despite their central role in supportive supervision, clinical mentoring, ownership, and sustainability of the CBNC program within the district health system. The study was conducted with participants who recently had been seeking care or provided services within the primary healthcare, mainly at health centers and health posts. Finally, although most of the findings are likely to reflect the situation in many Ethiopian rural settings, their transferability may be limited when contexts differ.

## Conclusion

Mothers’ poor knowledge about neonatal illnesses delayed care seeking, indicating the importance of reinforcing awareness creation on newborn danger signs. Lack of awareness and frequent closures of health posts minimized care seeking at health post. While at health centers, mothers experienced delayed care for their sick young infants, lack of physical examination of their sick young infants, and poor communication on findings and treatment. Lack of drugs and a weak referral system affected the care provided at health posts and health centers. Furthermore, district-level ownership of CBNC services affected the utilization and quality of care. This findings call for a holistic approach to enhance care for sick young infants in primary healthcare settings. Efforts should focus on equipping mothers and families with knowledge and resources about newborn danger signs and prompt care-seeking; enhancing healthcare providers’ capacity through in-service training to ensure facilities have adequate equipment, drugs, trained staff, and high-quality supportive supervision and clinical mentorship to ensure consistent availability of good-quality care. Finally, district-level ownership needs to be strengthened to ensure the sustainability of CBNC services. Overall this study emphasizes the importance of integrating demand-creation activities with strengthening the health system to ensure consistent service availability and quality care for sick young infants to reduce neonatal mortality.

## Supplementary Information

Supplementary material 1. S1: qualitative tools.

## Data Availability

The data and interview transcripts cannot be made publicly available due to confidentiality reasons. The transcripts did not include names of respondents. However, they included facility names and individual names like names of health extension workers and health center staff.

## Abbreviations

CBNC: Community-based newborn care
HEW: Health extension worker
IDI: In-depth interview
IMNCI: Integrated Management of Newborn and Childhood Illness
KII: Key informant interview
NGO: Non-governmental organization
PSBI: Possible serious bacterial infection
WHO: World Health Organization
