# Supplementary material for: Caregiver experiences and healthcare provider perspectives on managing sick young infants in primary care: a qualitative study in Awi Zone, Northwest Ethiopia
Source: Trop Med Health. 2025 Dec 11;54:7. doi: 10.1186/s41182-025-00873-8 (PMC12781729; doi:10.1186/s41182-025-00873-8)
Supplement: Supplementary file 1 — Supplementary material 1. S1: qualitative tools. [file 41182_2025_873_MOESM1_ESM.docx]

**S1: qualitative tool**

**In-depth interview with mothers of sick young infants, health center staff, and health extension workers. Key informant interview with health extension worker supervisors.**

**In-depth interview guide for mothers of sick young infants**

Participant code: |__|__|__|

Interviewer ID: |__|__|

Interview date ፡ |__|__/__|__/__|__|

Interview start time፡ _________

Interview end time: _________

| **Questions** | **Probing questions** |
| --- | --- |
| **Illness recognition** | |
| 1. Would you tell me about your last delivery?   (Ask the name of the last baby) | - Place of delivery - Overall process of delivery - If any complication, how was it managed |
| 1. How was the health of your newborn in the first two months after delivery? If illness was mentioned |  |
|  | Signs and symptoms noticed first  Perception on cause of illness? What makes you to think like that?  In your opinion how sick was your newborn? What made you to think that?  Age of the newborn at time of illness |
| **Preference of care (Care seeking pattern)** | |
| 1. After you know your baby was ill what did you do? | *Let the mother narrates the pattern of care seeking (from home to appropriate source of care)*  If nothing was done initially, what was the reason?  If care was provided at home,  Who provided the care  What was done  Reasons for the preferred care option  How did you feel about it? Why?  If they sought care other than appropriate source of care,  Place of care  Who provided care  What was done  Reasons for the preferred care option  How did you feel about it? Why?   - If they sought care from appropriate source of care,   - Place of care (home by HEWs/ health post/health center) |
| 3.1- How long did it take after recognition of signs of illness to care seeking at appropriate source of care? | - If immediately: what helped you to go soon? - If delayed: what were the challenge? |
| 3.2- Who was involved on deciding the preferred source/type of care? | - Process of decision making - Who took the final decision? - What things were considered to make the decision? - How do you feel about this process? |
| **Experience of care** | |
| 1. After deciding to seek care at the facility would you narrate me the steps you have gone through? | *Journey to health facility*   - Mode of transport to the facility and cost (Foot, vehicle,…) - Time taken to arrive, factors for the time taken - Experience during the journey (feelings and reasons)   *Process of care at health facility*   - Time gap between arrival at facility to care provision - How did you feel about it?   After you inter to the examination room:-   - Questions asked by the healthcare provider about the baby illness - What was done?(temperature, weight, breathing, cord, breast feeding status assessed) - Resources used to assess the baby (thermometer, weight scale, stopwatch, chart booklet, register) - Information told by healthcare provider about your baby’s illness - Medications provided (type, what were you told to do with the medication?)     *If referred,* Would you please tell me the referral process?   - Reason of the referral (Information given/ not given) - Referral accepted/ refused? Reasons for accepting or refusing - Feeling when referred. Reasons to feel like that - Means of transport? Any cost? What do you feel about it? - How long after referral did you go to the referral facility? Reasons to go soon or delay |
| 4.1- How did you describe the care you received for your sick baby? *(if referred ask at both the first facility and the referral facility)* | In terms of:   - The facility (infrastructure, drug, equipment..) - Healthcare providers (knowledge, skill, attitude, respect…) - If the answer is good or bad, reasons to say good/bad? |
| 4.2- How do you describe your satisfaction with the service you received for your sick baby? *(if referred ask at both the first and the referral facility)* | - If satisfied: What were the things that satisfied you? - If dissatisfied: What were the reasons for your dissatisfaction? |
| 1. How **do you monitor** your baby’s health once you returned back home? | - How does the healthcare provider tell you to provide the medication? How did you provide it? - If provided as ordered, what helped you? If not what was the challenge? - Did you let your baby finish the medication? Factors to finish/ not to finish   If the treatment was with injection:   - What was the process like for provision of injection? - Where was it done? For how long? - How do you feel about the process of medication provision? |
| 5.1- What was the outcome of your baby’s health? | - What do you feel about it? |
| 1. In your opinion what would have improved the care that was provided for your newborn? | - overall experience of care |
| 1. What do you suggest for friends/ family members if you saw same symptoms or a sick newborn again in the future? | - Reasons for the suggested options |
| 1. In your community where do mothers go if they got a sick baby? | - Reasons for the preferred care option - Enabling factors to seek care at the health facilities - Challenges to seek care at the facilities |
| 1. What kind of services did you got at health posts or from HEWs for young infants aged 0-2 months? | - Sick young infants curative care service? |
| 1. What do you suggest to overall care provided at health posts/ health centers to be improved? |  |
| 1. Anything you want to add? |  |
| 1. Finally I have some questions about you | 12.1- How old are you?  12.2- What is your and your husband education level?  12.3- Marital status?  12.4- How far is the nearest facility from your home? (on foot or car; in minute/Kilo meter)  12.5- Residence? Rural/Urban |
| **Summarize**  **Thank you for your participation and time** | |

**In-depth interview guide for health center staff**

Participant ID No |__|__|__|__|

Interviewer ID: |__|__|

Name of the health center: _________

Interview date: |__|__/__|__/__|__|

Interview start time: _____________

Interview end time:_____________

| **Questions** | **Probing questions** |
| --- | --- |
| 1. What is your role in this facility? | - Duration of work in under-five clinic - Experience on managing young infants |
| 1. Can you tell me about any training/ training update you may have had related to young infants care? | - How long ago, where, provider of training, number and type of trainees - Content, duration of training, method (practical/theoretical sessions) - Focus area of the training.(on identification and management of possible serious bacterial infection) |
| - 1. How do you describe your performance on managing sick young infants after the training? | - Knowledge and skill on management of possible serious bacterial infection - Confidence |
| - 1. Would you tell me what might have improved your training? |  |
| 1. In your stay in under-five clinic what kind of illness did you usually encounter in young infants? | - The most common and least common illnesses encountered |
| **Assessment and Case classification** | |
| 1. Remember the last time you see a sick young infant and would you please narrate the process of care provided?   **select a most recent possible serious bacterial infection case from register book and ask if she/he remember the case.* | - How the young infant come to health center (referred/directly)   *If come directly to the facility,*   - Signs and symptoms of the young infant - What was done next (Questions asked, what and/or how assessed) - Diagnosis - Feeling on examining the young infant, reasons to feel in such a way - Factors that helped to identify the illness/ diagnose - Challenges to identify/diagnose the illness infant illness?   *If referred from health post,*   - Reasons for referral - Sign and symptoms of the young infant - What was done next - How do you describe the referral process?/the performance of the health extension workers on: - Diagnosis of the young infant (diagnosis of the health extension worker related to the health center staff) - Any pre-referral antibiotics, describe - Referral slip (Information on referral slip) - Challenges noticed on health extension workers ability on identification of the case (if any) - Challenges you noticed on referral process (if any) - What do think should be done to solve these problems? |
| **Treatment of the sick young infant** | |
| 1. Now keeping this young infant in mind what happened after you made the diagnosis? | *If treated at that level*,   - Types of antibiotics provided (How antibiotics given, how often, for how long and where) - Factors allowed to provide appropriate treatment - Factors that prevented to provide appropriate treatment   *If referred to hospital,*   - How was the referral process?   *If pre-referral antibiotics given/not given*:   - Factors allowed to provide appropriate pre-referral antibiotics - Factors that prevented to provide appropriate pre-referral antibiotics   *If sent back to health post for completion of treatment,*   - When the baby sent back to health post? Reason - What was done (feedback, antibiotics)   *If nothing was done*, what was the reason? |
| 1. Thinking of the same young infant, now can you tell me what kind of care they received afterwards? | *If referred to hospital or sent back to health post*:   - Means of follow up   *If treated at that level*:   - Treatment adherence; How do you know their treatment adherence? - Outcome, mean to know the outcome (if unknown, reasons for unknown outcome) - Challenges for follow up and provision of treatment |
| **Perception of Health center staff on users experience** | |
| 1. How do mothers react with the service you provided for the baby? | *If treated at that level*,   - Reaction of the mother with the proposed management options   - How did you respond for this?  - How do these impacts your work?  *If referred to hospital or send back to health post*,   - Reaction of mother on referral or returning back to health post for treatment - How did you manage it? - Satisfaction of mothers; How are you able to gauge their satisfaction? - Reasons for satisfaction or lack of satisfaction? How does this affects your work? |
| 1. In your view what are the enabling factors to manage young infants with possible serious bacterial infection at your facility? | - Enabling factors to identify and classify   -Related to health work force, facility preparedness, capacity buildings, community perception/preference of care   - Enabling factors to treat   -Related to health work force, facility preparedness, capacity buildings, community perception/preference of care |
| 1. What do you think are the main challenges that the health center staff faced in managing possible serious bacterial infection in young infants at your facility? | - Challenges to identify and classify   -Related to health work force, facility preparedness, capacity buildings, community perception/preference of care  -How these challenges should be improved? By whom?   - Challenges to treat   -Related to health work force, facility preparedness, capacity buildings, community perception/preference of care  -How these challenges should be improved? By whom? |
| **Supportive supervision** | |
| 1. Can you tell me about the supportive supervision that is provided to you? **Think of the last supportive supervision you received* | - Time of last supportive supervision, provider of supervision - Frequency, way of supervision (one/two way), activities done during supervision (contents) observation/discussion related to possible serious bacterial infection) |
| - 1. How do these visits provide you with support? | - Ways to address gaps in your work - Challenges noticed on the supportive supervision |
| - 1. If you were a supervisor how will you conduct the supervision? |  |
| - 1. In your opinion how can the supervision be improved further to help you more on provision of quality care for sick young infants? |  |
| 1. Anything you want to add? |  |
| 1. Finally let me ask you about your background | - 1. Education background   2. Service year   3. Duration of work at under-five clinic   4. Profession   5. Age |
| **Summarize**  **Thank you for your participation and time** | |

**In-depth interview guide for health extension workers**

Participant ID No |__|__|__|__|

Interviewer ID: |__|__|

Name of the health post: _________

Interview date: |__|__/__|__/__|__|

Interview start time: _____________

Interview end time:_____________

| **Questions** | **Probing questions** |
| --- | --- |
| **Services provision for young infants** | |
| 1. How do you know if there is a newborn baby at your community? | - Time gap (how long after delivery) - What do you do then - Challenges and enabling factors |
| 1. After you know there is a newborn baby what kind of service do you provide for young infants 0-2 months of age? | - Types of newborn care services - Types of services with highest number   - Enabling factors   - Types of services with low number   -Reasons  -How it should be improved   - Services related to sick young infants - Places of service provision (facility/community) |
| 1. How do you describe the awareness of mothers on the types of services provided at health post/ by the HEWs? | - Means of awareness - Awareness related to sick young infants care - Challenges noticed - Suggestion to improve their awareness |
| 1. Can you tell me about any training you may have had related to young infants care? | - How long ago, where, number of trainees, training provider, language of training - Content, duration of training, method (practical and/or theoretical sessions) - Focus area of the training (on identification and management of possible serious bacterial infection; gentamicin skill) - Performance after the training (on: knowledge and skill on management of possible serious bacterial infection) |
| - 1. Would you tell me what might have improved your training? |  |
| 1. Would you please tell me how the HEWs find out when there is a sick young infant in your community? | - Source of information - What was done after being informed - Challenges and enabling factors |
| 5.1- Where do you think mothers mostly seek care for their sick young infants? | - Reasons |
| 1. In your view what are the enabling factors to provide care for young infants illnesses at your facility/ community? | - Related to health work force, facility preparedness, capacity buildings, community perception/preference of care |
| 1. What do you think are the main challenges that the health extension workers face in providing care for young infants illnesses at your facility/ community? | - Related to health work force, facility preparedness, capacity buildings, community perception/preference of care - How these challenges should be improved? By whom? - Suggestions |
| 1. In your opinion where do you think sick young infants should be managed? | - Reasons |
| **Supportive supervision and mentoring** | |
| 1. Can you tell me about the supportive supervision that is provided to you? **Think of the last supportive supervision you received* | - Time of last supportive supervision, supervision provider - Frequency, place of supervision (facility &/or community), way of supervision (one/two way), - Activities done during supervision (contents) related to:   - Observation/discussion  - Client consultation  - Observe records, supplies, medicines…  - Feedback |
| - 1. How do these visits provide you with support? | - Ways to address gaps in your work - Challenges noticed on the supportive supervision |
| - 1. If you were a supervisor how will you conduct the supervision? |  |
| - 1. In your opinion how can the supervision be improved further to help you more on provision of quality care for young infants? |  |
| 1. Can you think back to the last performance review and clinical mentoring meeting you attended and describe what took place? | - When, where, number and type of participants, - Focus of discussion during the meeting   - On identification and management of possible serious bacterial infection  - On your skill and confidence   - Overall performance review and clinical mentoring meeting schedule (frequency) |
| - 1. How do you describe the role of attending the performance review and clinical mentoring meeting relative to your work on young infants illness? | - Ways to address gaps in your work - Challenges noticed on the clinical mentoring meeting |
| - 1. Can you tell me how the performance review and clinical mentoring meeting can be improved further? |  |
| 1. Anything you want to add? |  |
| 1. Finally let me ask you about your background | - 1. What is your level of education?   2. How long have you work as health extension worker?   3. How long have you work at the current health post?   4. What is your position at the health post? (head /not)   5. How old are you? |
| **Summarize**  **Thank you for your participation and time** | |

**Key informant interview guide for health extension worker supervisors**

Participant ID No |__|__|__|

Interviewer ID: |__|__|

Name of the health center: _________

Number of catchment health posts: _________

Interview date: |__|__/__|__/__|__|

Interview start time: _____________

Interview end time: ______________

| **Questions** | **Probing questions** |
| --- | --- |
| 1. Would you tell me your role in this health center? | - Experience on young infants’ health care (work at under-five clinic) - Related to health extension workers supervision |
| 1. Can you tell me about any training you may have had related to young infants care? | - How long ago, where, provider of training, number and types of trainees, - Contents, duration of the training, method (practical/theoretical sessions) - Any orientation on how to supervise and support health extension workers?   -Related to possible serious bacterial infection management |
| - 1. Would you tell me what might have improved your training? |  |
| 1. Can you tell me specifically how you provide supervision for health extension workers? (*Think of the last supervision you provided and tell me about it)* | - Schedule - Activities done during supervision   (Discussion, observation, community consultation, feedback)   - Acceptance of HEWs on the supportive supervision |
| 3.1- In your opinion how do you see the role of supervision related to health extension workers performance on identification, classification and treatment of possible serious bacterial infection in young infants? |  |
| 1. In your view during supervision what are the challenges that health extension workers frequently face on managing possible serious bacterial infection in young infants? | - Reasons for these challenges - How should these challenges be improved |
| 1. How do you describe the knowledge and skill of health extension workers on management of possible serious bacterial infection in young infants? | - On identification of possible serious bacterial infection - On classification of possible serious bacterial infection - On treatment of possible serious bacterial infection |
| 1. How do you describe the preparedness of the health posts to provide service to manage sick young infants including possible serious bacterial infection? | - Related to health facility preparedness (infrastructure, drugs, equipment) |
| - 1. How do you think availability of drugs and equipment affect the management of possible serious bacterial infection in young infants? |  |
| 1. How do you describe the referral linkage between health posts and health centers? | - Feedback mechanism - Challenges seen during referral process   If challenges mentioned:   - How do you think these challenges will be improved? |
| 1. How does the follow-up of referred possible serious bacterial infection cases from health post conducted at health center? | - Enabling factors - Challenges |
| 1. What are the enabling factors in providing supportive supervision? |  |
| 1. What are the challenges to provide supportive supervision? |  |
| 1. As health extension workers supervisor what are your suggestions to improve the performance of the health extension workers? |  |
| 1. Anything you want to add? |  |
| 1. Finally let me ask you about your background | 13.1- Educational background  13.2- Profession  13.3- Service year   - 1. Duration worked as health extension worker supervisor   2. Age |
| **Summarize**  **Thank you for your participation and time** | |
